# Multidimensional gene search with Genehopper

**DOI:** 10.1093/nar/gkv511

**Published:** 2015-05-18

**Authors:** Matthias Munz, Sascha Tönnies, Wolf-Tilo Balke, Eric Simon

**Affiliations:** 1 Institute for Information Systems, Technische Universität Braunschweig, Braunschweig 38106, Germany; 2 Target Discovery Research, Boehringer Ingelheim Pharma GmbH & Co. KG, Birkendorfer Str. 65, Biberach (Riss) 88397, Germany

## Abstract

The high abundance of genetic information enables researchers to gain new insights from the comparison of human genes according to their similarities. However, existing tools that allow the exploration of such gene-to-gene relationships, apply each similarity independently. To make use of multidimensional scoring, we developed a new search engine named Genehopper. It can handle two query types: (i) the typical use case starts with a term-to-gene search, i.e. an optimized full-text search for an anchor gene of interest. The web-interface can handle one or more terms including gene symbols and identifiers of Ensembl, UniProt, EntrezGene and RefSeq. (ii) When the anchor gene is defined, the user can explore its neighborhood by a gene-to-gene search as the weighted sum of nine normalized gene similarities based on sequence homology, protein domains, mRNA expression profiles, Gene Ontology Annotation, gene symbols and other features. Each weight can be adjusted by the user, allowing flexible customization of the gene search. All implemented similarities have a low pairwise correlation (max *r*^2^ = 0.4) implying a low linear dependency, i.e. any change in a single weight has an effect on the ranking. Thus, we treated them as separate dimensions in the search space. Genehopper is freely available at http://genehopper.ifis.cs.tu-bs.de.

## INTRODUCTION

Emerging high-throughput technologies like next-generation sequencing (NGS) have led to a dramatic increase of descriptive and functional genetic information over the past decade, revealing gene properties such as gene and protein family, mRNA tissue distribution, gene functional or pathway membership. Many very powerful tools have evolved to explore this information, e.g. Ensembl, RefSeq, Homologene, UniProt, InterPro, Gene Ontology (GO), KEGG, REACTOME, GTExPortal, THE HUMAN PROTEIN ATLAS, or even meta-tools like STRING or MSigDB. Further processing of these properties into gene similarities enables the unbiased exploration of inter-gene relationships, representing a useful application in target discovery research.

Since in the present study we focus on human coding genes we use the term ‘gene’ as a generic term for a gene locus and the corresponding gene products, i.e. transcripts and proteins.

Several computational tools exist for finding gene relationships. The UCSC GeneSorter (1) (available at: https://genome.ucsc.edu/cgi-bin/hgNear) ranks genes from human, rat and mouse based on the similarity of expression profiles of the GNF Gene Expression Atlas, GO terms (2), the proximity in genome, the gene name and both structural and functional protein similarities. It provides additional filtering options for restricting the gene corpus, e.g. to a certain chromosomal position. EvoCor (3) (available at: http://pilot-hmm.vbi.vt.edu) ranks genes according to putative functional linkages with a gene of interest. This functional similarity is predicted from evolutionary information, i.e. sequence homology and gene expression data. It incorporates genes from all eukaryotic species in the NCBI database. LineUp (4) (available at: http://caleydo.org/projects/lineup) is another, generic approach which uses bar charts to rank items in an interactive way. It allows multiple heterogenous attributes, i.e. has no restrictions on certain scales or semantics. However, compared to the UCSC GeneSorter and EvoCor it is not query based, i.e. does not rank objects according to the similarity to a query (e.g. a gene) and therefore only provides limited possibilities for finding gene-to-gene relationships. Instead, it can rather be used as a tool in the field of gene prioritization.

In the tools described above the number of genetic similarities is small and/or each similarity is used independently without making use of multidimensional scoring. In this paper, we present a new search engine with a focus on human genes that incorporates multiple gene similarities into a weighted linear model. The weighted model allows the user to combine these similarities into a single ranking score according to the user's needs. Additionally, we put a strong emphasis on the speed and accuracy of the web based search and retrieval front-end. To integrate our tool into the landscape of public available resources, the search results links to external web sites such as Ensembl or PubMed. We chose the name Genehopper to clarify the ability of easy and fast navigating through the corpus of human genes.

## MATERIALS AND METHODS

**Figure 1.**
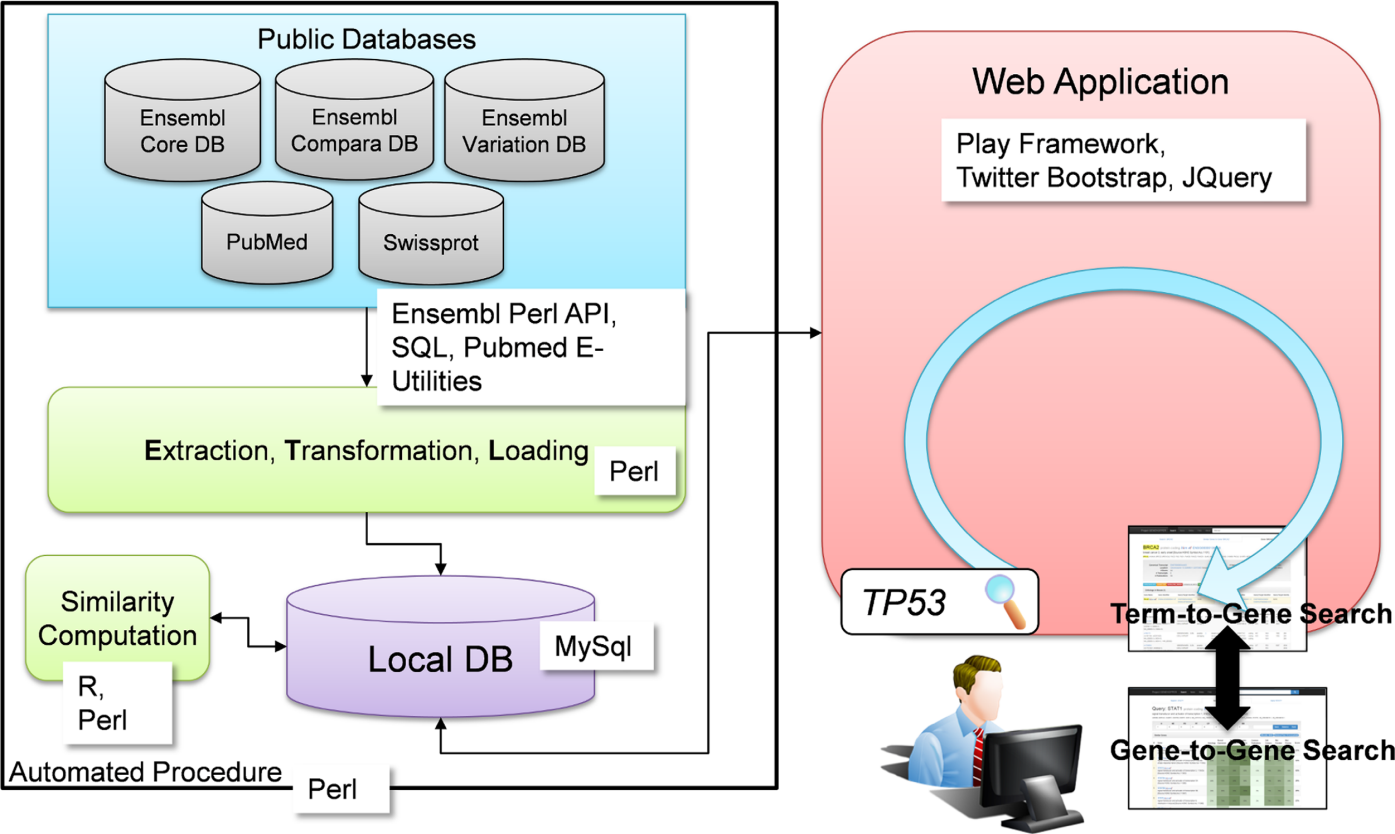
Data from various public databases is fetched to create a local and integrated database (left component). The web application of Genehopper is used to access the data by providing the search types term-to-gene search and gene-to-gene search (right component). The workflow on the web front-end is initiated by a text query, e.g. *TP53*.

The architecture of Genehopper consists of a data integration and a data application component (as seen on the left and right of Figure 1, respectively). Data from various public databases is extracted, transformed into the internal data model and finally loaded into a local MySQL database (ETL process). The ETL procedure is implemented in Perl and fully automated to allow regular updates.

### Data sources

Most of the data has been extracted from Ensembl (5) version 79 via Ensembl Perl API or SQL. From the Ensembl Core database we got information on positional data of genes, exons and transcripts, Ensembl identifier mappings to other databases such as InterPro, UniProt, RefSeq, EntrezGene, Gene Ontology, InterPro as well as external reference data including gene names, descriptions and synonyms. From the Ensembl Compara database we got information about paralogous and orthologous genes and the corresponding amino acid sequence based homologies. From the Ensembl Variation database (6) we got information concerning non-structural genetic variants including dbSNP identifier, synonyms and variant related citations. Data extraction from Ensembl Core and Ensembl Compara was done by using the Ensembl Perl API. For data from the Ensembl Variation database we chose SQL due to performance reasons. Abstracts of the cited publications were fetched from PubMed with the tool E-Utilities (documentation available at: http://www.ncbi.nlm.nih.gov/books/NBK25497). Protein feature information was fetched from the manually curated UniProtKB/Swiss-Prot database.

All semantic gene information has been compiled into a single MySQL table. For this table a full-text index was created to associate user query terms to target genes. An exemplary database table is shown in Supplementary Figure S1.

### Search engine - Concept and implementation

The web front-end application is implemented in Java and Javascript by utilizing the Play! Framework (available at: https://www.playframework.com), jQuery (available at: http://jquery.com) and Twitter Bootstrap (available at: http://getbootstrap.com). The term-to-gene search was realized using a two-step approach: (i) the user query terms are matched against the full-text index described above giving a set of hit genes that match the query. (ii) These hits are then ranked by the type of match e.g. a match to the HGNC symbol is ranked higher compared to a match to one of the synonyms or a substring match.

The gene-to-gene search is based on the ranked multidimensional gene similarities. In order to calculate an overall ranking score for each gene we use a linear weighed sum model according to:

(1) \begin{equation*} S = f(\vec x) = w_1 x_1 + w_2 x_2 + \cdots + w_n x_n \end{equation*}

*x* denotes the similarity vector of size *n* for a pair of anchor gene and result gene. Each similarity *x_i_* is multiplied with a weight *w_i_* to adapt the ranking. Result genes are ranked in descending order according to their overall ranking score which corresponds to the weighted sum of the pairwise similarities between the anchor gene and all other genes. To facilitate interpretation of the similarity values and the overall ranking score we constrain the normalization of the weights and similarities according to:

(2) \begin{equation*} \sum\nolimits_{i = 1}^n {w_i = 1} \end{equation*}

\begin{equation*} 0 \le w_i \le 1 \end{equation*}

\begin{equation*} 0 \le x_i \le 1 \end{equation*}

To improve the search performance we pre-calculated the similarities as part of the automated ETL procedure. Only the similarity values above zero are kept and stored to minimize the database size. Moreover for both the term-to-gene and the gene-to-gene search the result lists of the 100 most frequent queries are cached using the EHCache (available at: http://ehcache.org) which is part of the Play! framework.

### Gene similarities

Altogether we selected nine different gene and protein similarities which have been scored according to five different similarity measures (see Table 1) to be most suitable: (1) Accordingly, S_HOM_ corresponds to the protein sequence identity according to the Ensembl Compara database. For all other genes the homology similarity value is set to zero.

**Table 1.** Gene similarities used in the gene-to-gene search and the respective similarity measure

	Similarity	Data source	Similarity measure
1.	Homology (*S*_HOM_)	Ensembl Compara	Sequence identity
2.	InterPro protein domain (*S*_IPD_)	Ensembl Core	Cosine
3.	Gene variant related publications (*S*_VP_)	Ensembl Variation	Cosine
4.	Swiss-Prot protein feature (*S*_SPF_)	UniProtKB/Swiss-Prot	Cosine
5.	GO cellular component (*S*_CC_)	Ensemble Core	Resnik-BMA
6.	GO molecular function (*S*_MF_)	Ensemble Core	Resnik-BMA
7.	GO biological process (*S*_BP_)	Ensemble Core	Resnik-BMA
8.	Normal tissue expression profile (*S*_NEX_)	Human Protein Atlas	Spearman
9.	HUGO gene symbol (*S*_HGS_)	HGNC	Prefix distance

(2) *S*_IPD_ represents the similarity of protein domains and is derived from the presence or absence of InterPro protein domains in the corresponding Ensembl gene product entry. We applied cosine over the Jaccard or Dice measure because it produces higher similarity values for sets with very different sizes and a relatively big intersection.

(3) The similarity of publications related to sequence variants *S*_VP_ is calculated from publications that are associated with two given genes describing sequence variants in the gene loci. In most cases, the publications correspond to genome wide associations (GWA) of single nucleotide polymorphisms with phenotypic traits (GWAS). Again we applied cosine for the same reasons as mentioned before. The publication-variant associations were extracted from Ensembl Variation database which Ensembl in turn collected from dbSNP (7), EPMC (available at: http://europepmc.org) and UCSC (8).

(4) The similarity of protein features *S*_SPF_ was calculated from a binary vector of 24 protein features using Cosine again. These features are derived from the UniProtKB/Swiss-Prot database and include binary flags for subcellular localization, protein family membership and role in human diseases according to OMIM (Online Mendelian Inheritance in Man, http://omim.org/) (detailed list in Supplementary Table S1). Generally, we mapped each Ensembl gene to the corresponding UniProtKB/Swiss-Prot entry by cross-references of both databases to each other.

(5–7) Similarities of GO terms *S*_CC_, *S*_MF_, *S*_BF_ have been investigated extensively by others. Accordingly, we applied Resnik as single GO term similarity measure (9–12). As combination method to derive the overall similarity for two term sets we chose Best-Match Average. We used the R implementation in the GOSemSim package (13).

(8) For the similarity of gene expression in normal tissue *S*_NEX_ we used a recently published mRNA Seq panel of 27 normal tissues (14,15). As similarity measure we used Spearman rank correlation coefficient.

(9) The last similarity of gene symbol is computed from HUGO gene symbols from HGNC (16). As similarity measure we chose the prefix distance (Supplementary Figure S2).

## RESULTS AND DISCUSSION

Our goal with Genehopper was to provide a tool which combines multidimensional similarities of genes to systematically explore the neighborhood of human genes. Therefore, we subdivide the workflow on the web-based user interface into two steps. First, an initial anchor gene search by one or more keywords is performed (term-to-gene search). Next, the gene selected from the result list serves as the anchor gene for the multidimensional gene-to-gene search. In both steps, every gene that is shown can be loaded in a gene details view.

### Term-to-gene search

For selecting an anchor gene via the term-to-gene search, Genehopper is not restricted to certain input terms. Besides gene symbols and identifiers of Ensembl, UniProt, EntrezGene, RefSeq, Genehopper can also find genes by publication or variant identifiers, even unspecific vocabulary is handled. Typically, human users do not remember long and cryptical gene identifiers or database accession numbers from Ensembl, Refseq, UniProt & Co. In most cases, they use the gene symbol or a substring for searching. This could be demonstrated in a survey of user queries that have been sent to a gene search tool (17). Consequently, our term-to-gene ranking search is superior to many other search engines which require exact input queries in many cases (Supplementary Tables S2 and S3). Due to the optimized full-text index and caching mechanism the retrieval speed of the term-to-gene search is highly competitive with other search engines (Supplementary Figure S3).

The result page of term-to-gene search contains information about the result genes, including a gene symbol, hyperlinks to external references, a single-line description, synonyms and identifiers. To track how a result gene was derived from the query, information about the matched data model attributes is listed as well. Depending on the type of query match, a result gene is ranked higher, i.e. is more relevant to the user, or lower, i.e. is less relevant to the user. Strings or substrings in the result list that match the query terms are backed with yellow.

### Gene-to-gene search

**Figure 2.**
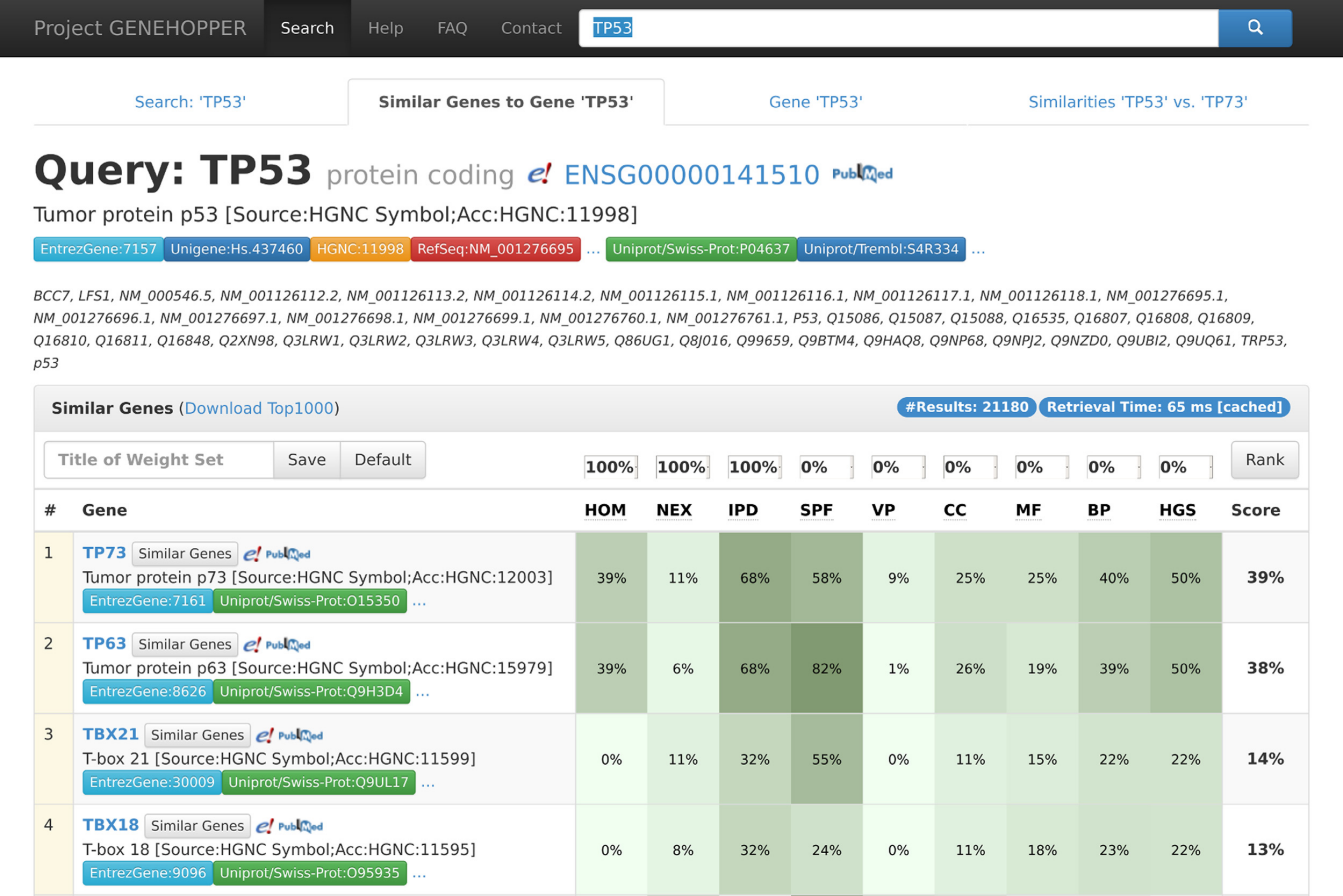
Result page of the gene-to-gene search with the exemplary query gene TP53. Each line in the result panel corresponds to a gene for which eight similarity scores and a ranking score is displayed. The ranking can be adjusted by reconfiguring the weights in the panel above the result list. Additional information about each similarity can be displayed by clicking on the similarity title.

Figure 2 shows the results page of the gene-to-gene search for one example, i.e. *TP53*. Genes in the result list are sorted descending according to their weighted pairwise similarities, i.e. ranking scores with respect to the anchor gene *TP53*.

The tool allows flexible customization of the gene search according to specific use cases by setting a pre-specified weight for each single similarity in the upper row of the search result panel. To facilitate the handling of the weight profiles, which by virtue of the restriction that the sum of all weights is one would contain odd values in the default case for nine similarities, each weight is multiplied by the number of similarities and displayed in percentage, leading to default values of one and 100 percent respectively. Adjusted weight profiles can be used to re-rank the result set (button on the right of weight settings) or saved to use them for future searches by setting a HTTP Cookie (buttons on the left of weight settings).

The similarity values are also shown in percentage and backed with a linear white-to-green color gradient. In contrast to other search engines, we employ a multidimensional similarity ranking with normalized ranking scores to illustrate the gene-to-gene relationships. The linear weighted similarity sum model allows a highly flexible ranking approach which could be useful for other bioinformatics applications, i.e. gene network construction, target pre-selection or candidate gene prioritization. EvoCor applies a graded sorting of the result genes, i.e. the result list is first sorted by sequence similarity and then by expression similarity. The UCSC GeneSorter only allows a sorting by a single similarity. When looking at individual gene similarities, UCSC GeneSorter handles all GO terms as a single set and measures the term overlap between genes. To expose a more finely granulated sight on the information in the Gene Ontology, we handle the three sub-ontologies molecular function, biological process and cellular component as separate similarities, not considering, e.g. process/function links between the sub-ontologies. LineUp allows the weighting of single attributes, but doesn't take advantage of similarity measures due to its non-query based architecture.

Moreover, we provide normalized (interval [0;1]) similarities and overall ranking scores in percentage quotation. This allows the user not only to infer similarity by assuming equal distance between every position, but to interpret each gene by a proper similarity value. We also evaluated the suitability of a machine learning algorithm for the personalization of the ranking (17). However, we did not follow this approach for the current study because different users prefer different rankings and even for a single user the preferred ranking can vary depending on the task.

To reduce the number of dimensions, we analysed and evaluated additional similarities and measures prior to the actual deployment of Genehopper. For this purpose we did a pairwise correlation of all similarities with all similarity values greater than zero (input data sizes in Supplementary Table S4). Table 2 shows the pairwise Pearson and Spearman correlation between the similarities (*P*-value < 2.2e−16 under the null hypothesis that the correlation is 0). Generally, both correlation coefficients have a very good agreement. This indicates that linear and monotonic trends are equally distinct. Regarding the correlation of similarities, the poorest correlation is observed between expression *S*_NEX_ and all other similarities (Pearson: [−0.04;0.08], Spearman: [−0.07;0.08]) except with *S*_HOM_ (Pearson: 0.27, Spearman: 0.21) as well as gene variant publication *S*_VP_ (Pearson: [−0.04;0.07], Spearman: [−0.16;0.03]) except with *S*_HOM_, *S*_IPD_, *S*_HGS_ (Pearson: [0.26;0.53], Spearman: [0.03;0.48]). Homology *S*_HOM_ has a relatively high positive correlation with all other similarities (correlation coefficient ≥ 0.21) except with *S*_HGS_ (Pearson: −0.17, Spearman: −0.15) and a maximum of 0.53 with *S*_VP_. For the other similarities, a somewhat higher correlation (Pearson: [0.16, 0.63], Spearman: [0.12, 0.63]) could be measured. Obviously, all similarities are not completely independent from each other. However, due to the relatively low overall correlations (max *r*^2^ = 0.4), we decided not to merge any of the similarities and to treat all of them as separate dimensions in the search space. The low correlations also imply that a change in weights has an effect to the ranking.

**Table 2.**
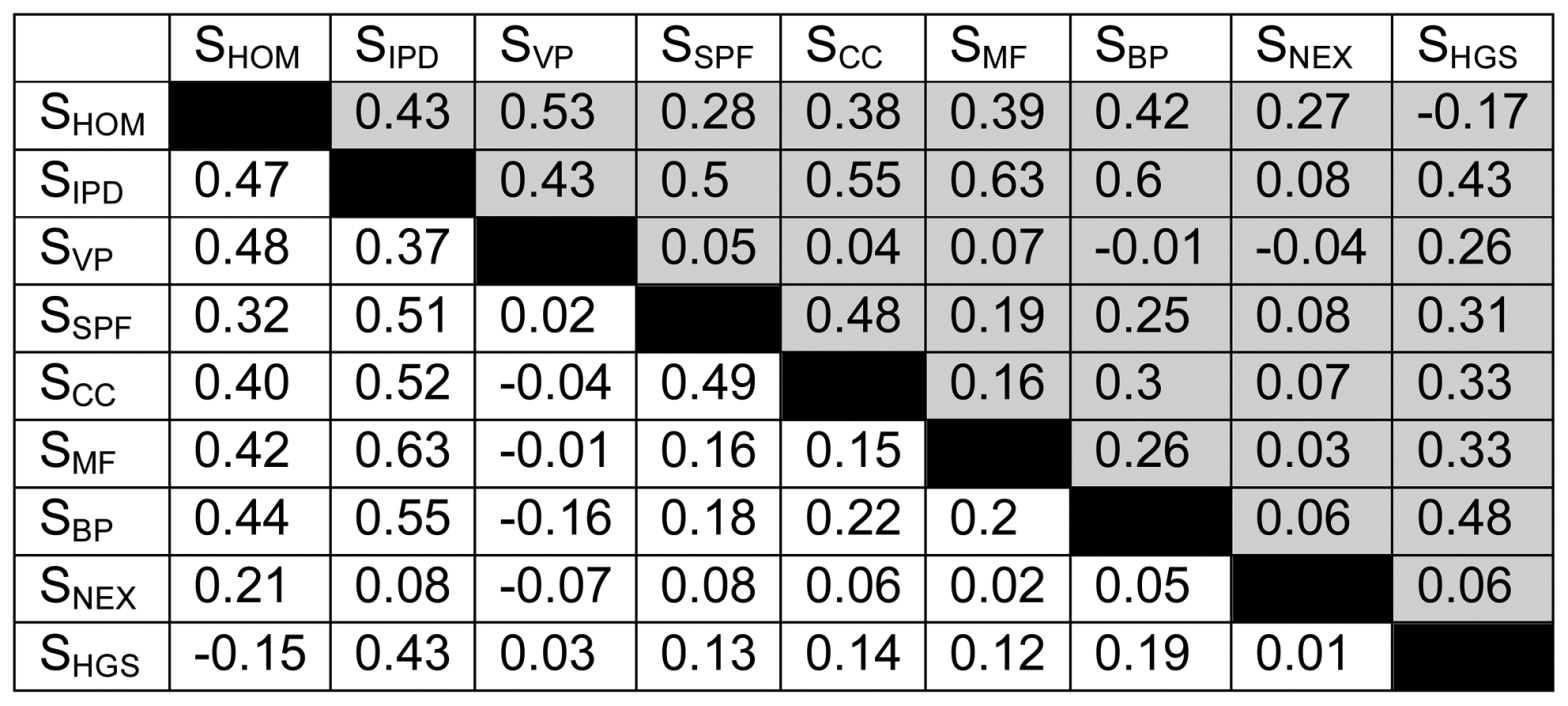
(Grey) Pearson and (white) Spearman correlations for each pair of the nine gene similarities

In order to gather a deeper knowledge of gene-to-gene relationships, we provide raw data from which the similarities were calculated by linking to a similarities page for each pair of anchor gene and result gene (link on similarity values). For further analysis with other tools the raw data for a pair of genes as well as the similarity values of the top 1000 result genes can be downloaded as text files.

## CONCLUSION AND OUTLOOK

Genehopper is a high performant search engine that extends the set of tools to explore human gene-to-gene relationships by a large number of gene similarities and the possibility to combine them into a multidimensional scoring model which facilitates the interpretation and increases the information content of rankings. Practically, the tool represents a powerful entry point to explore the molecular data and neighborhood of a given human gene or protein within the tool and by using the linked out resources. This will be useful for example to quickly evaluate functional similarities within larger gene families or to get a first insight into the function of genes where almost nothing is known from the literature.

To ensure relevance and reliability, the automated ETL procedure will be used to update the database regularly according to the update frequency of Ensembl (approximately twice per year) and also to include additional similarities which have not yet been included in the weighted ranking approach, i.e. curated pathway information, protein-protein interactions and genomic coordinates. Additionally, it would be very useful to provide a human readable summary of the similarity data, i.e. GO and InterPro names in addition to IDs, a visualization of the expression data (‘Electronic Northern Blot’) and the subcellular localization as well as the functional gene and protein information. Another potential direction for the future development of Genehopper is represented by personalized ranking. This could allow, i.e. optimized adjustment of the similarity weights for individual users. Furthermore, this would open the possibility to build up social network platforms for scientific groups which work on similar molecular targets, pathways or diseases. We tested already machine learning tools to predict the weight preferences of individual users (17). However, such an approach will heavily rely on a large user community.

## SUPPLEMENTARY DATA

Supplementary Data are available at NAR Online.

SUPPLEMENTARY DATA
