# Supplementary material for: Multidimensional gene search with Genehopper
Source: Nucleic Acids Res. 2015 May 18;43(Web Server issue):W98–W103. doi: 10.1093/nar/gkv511 (PMC4489220; doi:10.1093/nar/gkv511)
Supplement: SUPPLEMENTARY DATA [file supp_43_W1_W98__index.html]

Multidimensional gene search with Genehopper — Multidimensional gene search with Genehopper — SUPPLEMENTARY DATA 

# Multidimensional gene search with Genehopper

## SUPPLEMENTARY DATA

- SUPPLEMENTARY DATA
