# Supplementary material for: Multidimensional gene search with Genehopper
Source: Nucleic Acids Res. 2015 May 18;43(Web Server issue):W98–W103. doi: 10.1093/nar/gkv511 (PMC4489220; doi:10.1093/nar/gkv511)
Supplement: SUPPLEMENTARY DATA [file supp_gkv511_nar-00291-web-b-2015-File006.pdf]

Supplementary Information

Gen-Id	Attribut 1	Attribut 2	Attribut 3	Attribut 4	…	Attribut m
1	ENSG0000022397 2	ENSG00000223972, 100287596, 100287102, 37102, DDX11L1_001, DDX11L1_002, DDX11L1_202, DDX11L1_201, OTTHUMG00000000961, OTTHUMT00000362751, OTTHUMT00000002844, NR_046018, NR_051986, uc001aaa_3, Hs_714157, Hs_618434	DDX11L5, DDX11L1, NR_046018_2, NR_051986_1, DDX11L1_002, DDX11L1_001	DEAD/H (Asp_Glu_Ala_Asp/His) box helicase 11 like 5, DEAD/H (Asp_Glu_Ala_Asp/His) box helicase 11 like 1, Homo sapiens DEAD/H (Asp_Glu_Ala_Asp/His) box helicase 11 like 1 (DDX11L1), non_coding RNA_, Homo sapiens DEAD/H (Asp_Glu_Ala_Asp/His)		rs58108140, rs189107123, rs180734498, rs144762171, rs151276478
…						
n						

Supplementary Figure 1. Exemplary layout of the dedicated database table that is used in the term-to-gene search.

$$\boldsymbol{PrefixDistance(s,t) = length(s) + length(t) - 2CommonPrefix(s,t)}$$

Supplementary Figure 2. We used the prefix distance as string metric to compare pairs of gene symbols s and t.

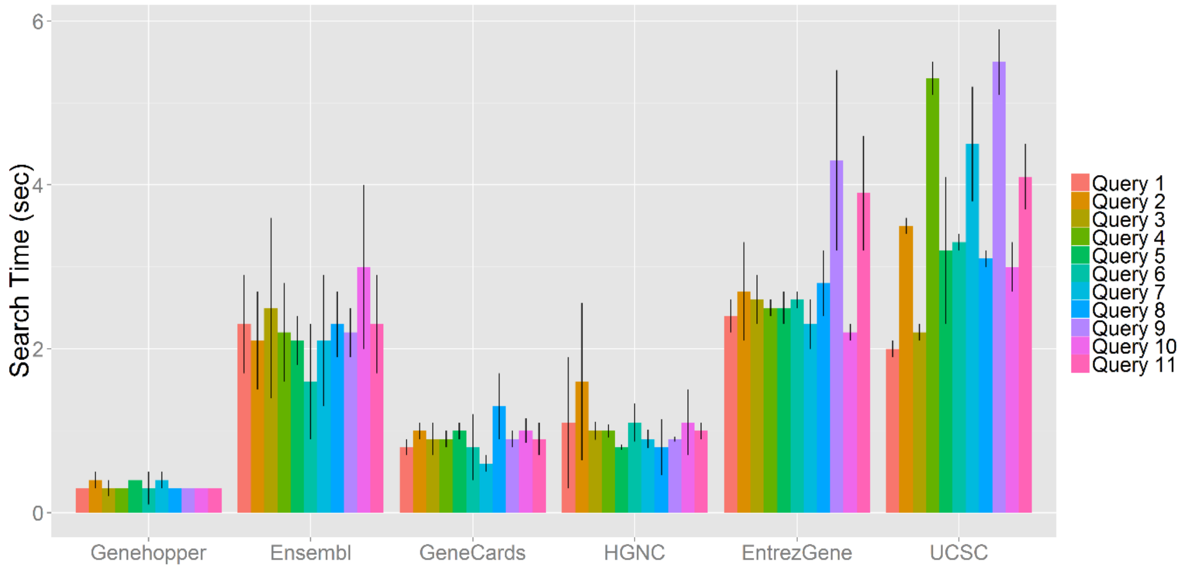

Supplementary Figure 3. Search time for Genehopper and five other search engines we considered in our performance test. We tested with eleven queries (1: CCR2; 2: PPARG; 3: CCN1; 4: RS1; 5: PCSK1; 6: Cannabinoid; 7: Cann; 8: Narcolepsy; 9: 100287102; 10: rs144303289; 11: ENSG00000069812) and 10 repeats per query in the Google Chrome Browser.

#	Label	Computed by UniProt Feature
1	Transmembrane	Presence of transmembrane domains
2	Signal	Presence of signal domains
3	Lipid	Presence of lipid anchors
4	Transcription Factor	Transcription Factor (presence of domains like IPR002070, …)
5	NHR	Nuclear hormone receptor (presence of IPR000536)
6	NOR	Nuclear orphan receptor (presence of IPR003070)
7	Ionchannel	Ion Channel (descripition; presence of domains like IPR000272, …)
8	GPCR	Class A G-Protein coupled receptor (presence of IPR000276)
9	Enzyme	Enyzme (field "EC" is defined)
10	Kinase	Kinase (EC number 2.7.x)
11	Protease	Protease (EC number 3.4.x)
12	Phosphatase	Phosphatase (EC number 3.[1|5].x)
13	PDE	PDE (ID PDE* or presence of IPR002073)
14	Disease	Disease related (presence of comment "Disease")
15	Monogenetic Disease	Monogenetic disease (Disease comment like "is a cause of …")
16	OMIM	Has reference to OMIM Gene-Phenotype relationshiop (UniProt)
17	Cytoplasm	Subcellular location in Cytoplasm (Comment)
18	Golgi	Subcellular location in Golgi apparatus (Comment)
19	Membrane	Subcellular location in Membane (Comment)
20	Mitochondrion	Subcellular location in Mitochondrion (Comment)
21	Nucleus	Subcellular location in Nucleus (Comment)
22	Secreted	Secreted (Comment)
23	Ubiquitome	Ubiquitome E3 or DUB (presence like IPR001841 or IPR001578, …)
24	Epigenome	Epigenome (DNA [Methyl|Acetyl]ation / De-acetylation)

Supplementary Table 1. 24 Gene protein product features vectors were computed according to the presence (value = 1) of absence (value = 0) of UniProtKB/Swiss-Prot Annotations (sequence related: 1-3; family related 4-13, 23, 24; disease related 14-16; sub-cellular localization 17-22). These vectors represent the data from which the similarity S_{SPF} was calculated.

No.	Query	Query Type	Expected Gene
1	CCR2	Gene Symbol	CCR2
2	PPARG	Gene Symbol	PPARG
3	CCN1	Gene Symbol	CYR61
4	RS1	Gene Symbol	RS1
5	PCSK1	Gene Symbol	PCSK1
6	Cannabinoid	Receptor Family, Gene Name	CNR1, CNR2
7	Cann	Substring of 'Cannabinoid'	CNR1, CNR2
8	Narcolepsy	Phenotype	MOG, HCRT
9	100287102	Entrez Gene ID	DDX11L1
10	rs144303289	dbSNP ID	RASGRP1
11	ENSG00000069812	Ensembl ID	HES2

Supplementary Table 2. Queries and the expected gene that we used for the comparison of the ranking quality of the Genehopper's term-to-gene search with other search engines. The results are shown in Supplementary Table 4.

No	Query	Genehopper	Ensembl	GeneCards	HGNC	EntrezGene	UCSC
1	CCR2	1	1	1	1	1	1
2	PPARG	1	1	1	1	1	1
3	CCN1	1	1	1	2	1	2
4	RS1	1	1	1	1	1	1
5	PCSK1	1	1	1	1	1	1
6	Cannabinoid	1; 2	1; 2	1; 2	1; 2	1; 2	1; 2
7	Cann	1; 2	1 [a]	-	-	-	1; 2 [b]
8	Narcolepsy	1; 2	1; 2	7; 15	-	10; 14	1; 2
9	100287102	1	-	1	1	1 [e]	-
10	rs144303289	1	[c]	1	-	[c]	[c]
11	ENSG00000069812	1	1	1	1	1 [d]	[d]

- gene not listed in result set

[a] CNR2 not in result set

[b] in category of RefSeq genes

[c] no gene assignment

[d] no name resolution

[e] immedidate forwarding to gene page

Supplementary Table 3. Comparison of the ranking quality for 11 queries that were applied to the search engines of Genehopper, Ensembl, GeneCards, HGNC, EntrezGene and UCSC by comparing the ranking positions of the expected genes in the respective search results. For queries 6, 7 and 8 two genes are expected, thus two ranking positions are shown. The expected genes are listed in Supplementary Table 3.

	S_{HOM}	S_{IPD}	S_{VP}	S_{SPF}	S_{CC}	S_{MF}	S_{BP}	S_{NEX}	S_{HGS}
S_{HOM}		5E+04	2E+03	3E+04	4E+04	5E+04	4E+04	4E+04	1E+06
S_{IPD}	5E+04		7E+03	1E+06	2E+06	2E+06	2E+06	2E+06	5E+05
S_{VP}	2E+03	7E+03		1E+05	2E+05	2E+05	2E+05	3E+05	3E+04
S_{SPF}	3E+04	1E+06	1E+05		6E+07	5E+07	6E+07	7E+07	5E+06
S_{CC}	4E+04	2E+06	2E+05	6E+07		8E+07	1E+08	1E+08	8E+06
S_{MF}	5E+04	2E+06	2E+05	5E+07	8E+07		9E+07	1E+08	7E+06
S_{BP}	4E+04	2E+06	2E+05	6E+07	1E+08	9E+07		1E+08	7E+06
S_{NEX}	4E+04	2E+06	3E+05	7E+07	1E+08	1E+08	1E+08		1E+07
S_{HGS}	1E+06	5E+05	3E+04	5E+06	8E+06	7E+06	7E+06	1E+07	

Supplementary Table 4. Size of input data to compute pairwise correlation between similarities. (Grey) Pearson, (White) Spearman.
